# Effect of Standard vs Intensive Blood Pressure Control on Cerebral Blood Flow in Small Vessel Disease: The PRESERVE Randomized Clinical Trial

**DOI:** 10.1001/jamaneurol.2017.5153

**Published:** 2018-03-05

**Authors:** Iain D. Croall, Daniel J. Tozer, Barry Moynihan, Usman Khan, John T. O’Brien, Robin G. Morris, Victoria C. Cambridge, Thomas R. Barrick, Andrew M. Blamire, Gary A. Ford, Hugh S. Markus

**Affiliations:** 1 Stroke Research Group, Department of Clinical Neurosciences, University of Cambridge, Cambridge, United Kingdom; 2 St George’s National Health Service Healthcare Trust, London, United Kingdom; 3 Department of Psychiatry, University of Cambridge, Cambridge, United Kingdom; 4 Institute of Psychiatry, Psychology & Neuroscience, King’s College London, London, United Kingdom; 5 Molecular and Clinical Sciences Research Institute, St George’s, University of London, London, United Kingdom; 6 Magnetic Resonance Centre, Institute of Cellular Medicine, Newcastle University, Newcastle upon Tyne, United Kingdom; 7 Oxford University Hospitals National Health Service Foundation Trust, Oxford, United Kingdom

## Abstract

**Importance:**

Blood pressure (BP) lowering is considered neuroprotective in patients with cerebral small vessel disease; however, more intensive regimens may increase cerebral hypoperfusion. This study examined the effect of standard vs intensive BP treatment on cerebral perfusion in patients with severe small vessel disease.

**Objective:**

To investigate whether standard vs intensive BP lowering over 3 months causes decreased cerebral perfusion in small vessel disease.

**Design, Setting, and Participants:**

This randomized clinical trial took place at 2 English university medical centers. Patients were randomized via a central online system (in a 1:1 ratio). Seventy patients with hypertension and with magnetic resonance imaging–confirmed symptomatic lacunar infarct and confluent white matter hyperintensities were recruited between February 29, 2012, and October 21, 2015, and randomized (36 in the standard group and 34 in the intensive group). Analyzable data were available in 62 patients, 33 in the standard group and 29 in the intensive group, for intent-to-treat analysis. This experiment examines the 3-month follow-up period.

**Interventions:**

Patients were randomized to standard (systolic, 130-140 mm Hg) or intensive (systolic, <125 mm Hg) BP targets, to be achieved through medication changes.

**Main Outcomes and Measures:**

Cerebral perfusion was measured using arterial spin labeling; the primary end point was change in global perfusion between baseline and 3 months, compared between treatment groups by analysis of variance. Linear regression compared change in perfusion against change in BP. Magnetic resonance imaging scan analysis was masked to treatment group.

**Results:**

Among 62 analyzable patients, the mean age was 69.3 years, and 60% (n = 37) were male. The mean (SD) systolic BP decreased by 8 (12) mm Hg in the standard group and by 27 (17) mm Hg in the intensive group (*P* < .001), with mean (SD) achieved pressures of 141 (13) and 126 (10) mm Hg, respectively. Change in global perfusion did not differ between treatment groups: the mean (SD) change was −0.5 (9.4) mL/min/100 g in the standard group vs 0.7 (8.6) mL/min/100 g in the intensive group (partial η^2^, 0.004; 95% CI, −3.551 to 5.818; *P* = .63). No differences were observed when the analysis examined gray or white matter only or was confined to those achieving target BP. The number of adverse events did not differ between treatment groups, with a mean (SD) of 0.21 (0.65) for the standard group and 0.32 (0.75) for the intensive group (*P* = .44).

**Conclusions and Relevance:**

Intensive BP lowering did not reduce cerebral perfusion in severe small vessel disease.

**Trial Registration:**

isrctn.org Identifier: ISRCTN37694103

## Key Points

**Question:**

Does intensive blood pressure lowering cause hypoperfusion in severe cerebral small vessel disease?

**Findings:**

This randomized clinical trial used arterial spin labeling to examine the effect of standard (n = 33) vs intensive (n = 29) blood pressure treatment regimens on cerebral blood flow in patients with severe small vessel disease over 3 months. Change in whole-brain cerebral blood flow did not significantly differ between standard vs intensive groups.

**Meaning:**

Intensive blood pressure lowering did not cause hypoperfusion in severe cerebral small vessel disease.

## Teaser

This randomized clinical trial investigates whether standard vs intensive blood pressure lowering over 3 months causes decreased cerebral perfusion in small vessel disease.

## Introduction

Cerebral small vessel disease (SVD) causes 20% of all ischemic strokes^1^ and is the most common cause of vascular cognitive impairment.^2^ It is thought that a diffuse arteriopathy of the cerebral small vessels results in hypoperfusion and impaired cerebral autoregulation, with subsequent ischemia leading to white matter hyperintensities (WMHs) visible on T2-weighted magnetic resonance imaging (MRI), as well as lacunar infarction.^2^

Hypertension is the most important risk factor for SVD.^3^ Prior studies have reported that more intensive blood pressure (BP) lowering (typically targeting a systolic BP of approximately 120 mm Hg compared with the standard target of 140 mm Hg) is associated with a reduction in cardiovascular events in primary prevention^4^ and with decreased recurrent stroke risk in secondary prevention in unselected patients with stroke.^5^ Such approaches may also be beneficial in SVD, but concern has been raised that overzealous BP lowering could exacerbate hypoperfusion,^6^ extending brain injury.

Studies^7,8^ have shown reduced cerebral blood flow (CBF) in the white matter (WM) (and normal-appearing WM [NAWM]) of patients with SVD and have shown that the degree of hypoperfusion correlates with the severity of WMHs, consistent with a causal role in the disease pathogenesis. It has also been demonstrated that the limits of autoregulation are shifted upward as a consequence of structural vascular adaptation in individuals with chronic hypertension.^9^ This evidence has led to the hypothesis that a higher systemic BP is required to maintain adequate CBF in SVD and that excess BP lowering may increase ischemia and worsen WMHs and cognition.^10^

However, a 2013 study^11^ demonstrated that intensive BP lowering in elderly patients with hypertension without stroke was associated with increased CBF, hypothetically due to a reshifting of the cerebral autoregulatory curve. In addition, a large trial on SVD showed a trend toward reduced recurrent stroke with intensive BP lowering.^12^ However, many patients in that study had isolated lacunar infarction without WMHs. Whether intensive BP lowering may be associated with worse outcome in patients with severe SVD and confluent WMHs, which is the group in whom severely reduced CBF and autoregulation have been reported, remains uncertain. To address this question, we performed a randomized clinical trial to investigate if standard vs intensive BP lowering in patients with lacunar stroke and confluent WMHs has different effects on CBF over 3 months.

## Methods

### PRESERVE Study

The PRESERVE study is an ongoing, 2-year, multicenter, randomized clinical trial that tests standard vs intensive BP treatment regimens in patients with severe SVD on outcomes of WM disease (assessed by diffusion-tensor imaging) and cognition. This article presents a nested substudy that investigates CBF change over the first 3 months of the trial. The full protocol can be found in the Supplement.

### Population

Screening by an experienced neurologist (B.M., U.K., or H.S.M.) ensured that all participants had MRI-confirmed clinical lacunar stroke, confluent WMHs graded as 2 or higher on the Fazekas Scale,^13^ hypertension, and no stroke within 3 months before study commencement. Detailed inclusion and exclusion criteria have been described previously.^14^ All patients gave written informed consent. The study was approved by the Harrow National Research Ethics Service committee and is registered with the UK Clinical Research Network.

A power calculation using arterial spin labeling (ASL) data^15^ from elderly controls showed that the planned sample size of 60 was powered at 0.9 to detect a 24% reduction in WM CBF and a 25% reduction in gray matter (GM) CBF (*P* = .01). Seventy patients with hypertension were recruited at 2 English university medical centers (41 at site 1 [St George’s National Health Service Healthcare Trust] and 29 at site 2 [Newcastle Hospitals National Health Service Foundation Trust]).

### Clinical Assessments

A neurologist or stroke physician (B.M., U.K., or other nonauthors) examined all participants. Cerebrovascular risk factors were recorded. At each participant visit (described below in the Trial Design, Randomization, and Treatment subsection), adverse events (AEs) and serious AEs (SAEs) were recorded. In addition to any general problems that had been experienced (eg, swollen ankles), participants were asked specifically about any falls or postural related dizziness that had occurred since the last visit. Any events reported were classified by likelihood of having been caused by a study drug (rated as none, remote, possible, probable, or definite) and classed as falls, postural related dizziness, or other.

### Trial Design, Randomization, and Treatment

The PRESERVE study has a parallel trial design. After recruitment, participants were randomized (stratified via site) to either the standard (systolic BP target, 130-140 mm Hg) or intensive (systolic BP target, <125 mm Hg) treatment group (aiming for a systolic BP difference between treatment groups of ≥15 mm Hg) in a 1:1 ratio via a centralized, online system (based at the Mental Health and Neurosciences Clinical Trials Unit, King’s College London) by a research nurse or physician, who then allocated the participant to the assigned group. No changes were made to the methods or trial outcomes after trial commencement. There were no interim analyses or stopping guidelines. Treatment allocation was known to participants and clinical staff (B.M., U.K., and other nonauthors), but analysis of data was performed masked.

Assessments in clinic were performed at baseline, 4 weeks, and 12 weeks. Additional clinic or telephone check-ups were performed as necessary. Participants took daily sitting readings with home BP monitors for at least 3 days before each check-up. During clinic visits, systolic and diastolic BP was measured in a sitting position 3 times after a 10-minute rest period in a quiet room. The recorded BP was the mean of the second and third measures. At each check-up, if the participant’s BP was above his or her study target (and provided that hypotensive symptoms did not prevent it), an increase in antihypertensive medication was prescribed. Results presented and used in the analysis were clinic BP readings.

Treatment algorithms for standard and intensive BP lowering protocols consistent with the British Hypertension Society/National Institute for Health and Care Excellence guidance on drug treatment of hypertension were used for reference.^16^ Final treatment decisions were made by the local study principal investigator (U.K. at site 1 and G.A.F. at site 2). No treatment changes were made for any participant before he or she had a baseline MRI scan.

### End Points

The primary end point was change in whole-brain CBF. Secondary end points were change in GM, WM, and NAWM CBF.

### MRI Protocol

Site 1 used a 3-T Achieva TX imaging system (Philips Medical Systems), while site 2 initially used a 3-T Achieva (n = 24) and then a 3-T Achieva TX (n = 5) after an upgrade (all participants received both scans on the same scanner). At baseline, the protocol included 3-dimensional (3-D) T1-weighted (T1W) and 2-dimensional fluid-attenuated inversion recovery (FLAIR) and pulsed ASL sequences. The T1W and ASL sequences were repeated at the 3-month time point. Details of each sequence are given below.

Sagittal 3-D T1W images included turbo field echo, voxel size of 1-mm isotropic, field of view (FOV) of 240 × 170 mm^3^, and repetition time/echo time of 8.27/4.61 milliseconds (site 1) and 9.81/4.60 milliseconds (site 2). Axial FLAIR included voxel size of 0.48 × 0.48 × 3 mm^3^, FOV of 230 × 230 mm^2^, 57 slices, repetition time/echo time of 11 000/120 milliseconds, and inversion time of 2800 milliseconds.

Axial pulsed ASL included FOV of 256 × 256 mm^2^, 10 slices, and inversion time at the most inferior slice of 1500 milliseconds. Site 1 used echo-planar imaging-based signal targeting by alternating radiofrequency pulses (EPISTAR),^17^ with a voxel size of 3.2 × 3.2 × 6 mm^3^, repetition time/echo time of 4000/27 milliseconds, 40° flip angle, and 120 tag/control pairs. Site 2 used flow-sensitive alternating inversion recovery (FAIR),^18^ with a voxel size of 4 × 4 × 6 mm^3^, repetition time/echo time of 2500/12.59 milliseconds, 90° flip angle, and 100 tag/control pairs.

Additional images were acquired for the ASL sequences. These included M0 and Geo EPI images for EPISTAR (voxel size of 3.2 × 3.2 × 6 mm^2^, FOV of 256 × 256 mm^2^, 10 slices, 90° flip angle, 1 acquisition, and repetition time/echo time of 15 000/27 milliseconds for M0 and 500/18 milliseconds for Geo) and sets of 6 acquisitions at inversion times of 900, 1200, 1800, 2100, and 2400 milliseconds for FAIR (same acquisition as the main sequence).

To ensure standardization, 4 healthy individuals underwent the full imaging protocol at each site before study commencement. The ASL data were preprocessed using standard techniques to create flow-weighted images, the histograms of which were examined, and consistent results between sites were found.

### Data Processing

#### ASL Preprocessing

Arterial spin labeling is an MRI technique that quantifies CBF without exogenous contrast agent. It instead uses the difference in signal between images where blood has or has not been magnetically tagged and converts this into CBF (in milliliters per minute per 100 g) via a model that takes into account variables such as the efficiency of the magnetic tag. It shows good agreement with contrast-enhanced MRI in populations with acute stroke^19^ and is considered a robust method of measuring CBF.^20^

The ASL image series were motion corrected using automated image registration (http://www.loni.usc.edu/Software/AIR).^21^ The difference in signal between average tag and control images was calculated, creating the difference image (dm). All tag/control images were averaged to create a magnitude image (M). The proton density (M0) image was acquired differently between the 2 sequences: for EPISTAR, the M0 image acquired during scanning was used, while for FAIR it was generated by fitting of the inversion recovery curve (modeled from the additional inversion time acquisitions). The dm/M0 images were coregistered to the M image using Statistical Parametric Mapping 8 (SPM8) (http://www.fil.ion.ucl.ac.uk/spm/) “coreg.”^22^

#### Region-of-Interest Generation

The WMHs were marked on the baseline FLAIR images as previously described.^14^ The T1W scans were intensity nonuniformity corrected (using N4ITK^23^), tissue segmented (using “Segment” from Statistical Parametric Mapping 12b), and registered to their time point respective ASL M image (using Oxford Centre for Functional MRI of the Brain’s [FMRIB’s] Linear Image Registration Tool, FMRIB’s Linear Image Registration Tool,^24^ and a part of the FMRIB Software Library^25^; for site 2, the registration was via the Geo image to improve registration success). FLAIR scans were registered to T1W images. Lesion masks and the segmented tissue probability maps (TPMs) were transformed into ASL space using these registrations (concatenated where necessary).

The transformed tissue probability maps were thresholded (at 0.5), binarized, and with the lesion masks used to create final masks of whole brain, GM, all WM, and NAWM. After visual inspection, any voxels that were outside of the brain (as defined by the M image) were removed from the masks.

#### CBF Calculation

The estimated signal in M0 images caused by cerebrospinal fluid partial volume was subtracted from the images. Cerebral blood flow was then calculated^26^ using MATLAB (version R2015a 8.5.0; MathWorks) (https://uk.mathworks.com/), where blood longitudinal relaxation time is 1550 milliseconds,^18^ blood tissue partition coefficient is 0.98 mL/g,^27^ and inversion time of the most inferior slice is 1500 milliseconds. Other variables differed between sequences. For FAIR, slice timing delay is 51 milliseconds, inversion efficiency (α) is 0.99, and transit delay (∆*t*) is 458 milliseconds. For EPISTAR, slice timing delay is 41 milliseconds, α is 0.9, and ∆*t* is 877.5 milliseconds. The CBF maps were calculated for each slice (accounting for timing delay), with total CBF for each tissue being an average of CBF across slices, weighted by the volume per slice.

### Statistical Analysis

Analyses were performed using SPSS (version 23; IBM Corporation). Variable distribution was measured by visual inspection and Kolmogorov-Smirnov test. Key demographic variables and risk factors were compared between treatment groups by independent *t* test, Mann-Whitney test, and χ^2^ test as appropriate. The number of AEs whose likelihood of being caused by the study drug had been deemed as possible/probable/definite was compared between treatment groups by Mann-Whitney test. Baseline CBF values for whole brain were compared by independent *t* test between sites to test for any site-related differences. These analyses were conducted using all available data, while all subsequent analyses considered only participants who returned usable ASL data sets from both time points.

Systolic and diastolic BP was compared between time points by paired *t* tests within each treatment group. Change in systolic and diastolic BP (ie, 3-month minus baseline BP) was compared by independent *t* tests between treatment groups.

### End Point Analyses

Analyses were performed on an intent-to-treat basis, were 2 tailed with α = .05, and controlled for site. Change in CBF (ie, 3-month minus baseline CBF) was compared between treatment groups using univariate analysis of variance (ANOVA). The Bayes factor^28^ was calculated for the whole-brain CBF model as an additional means of estimating the strength of the primary end point finding.

Further secondary analyses repeated the above ANOVA models but only included intensive group participants who reached their target vs standard group participants whose 3-month systolic BP exceeded 130 mm Hg (thus controlling for participants whose final BP may undermine study assumptions). Finally, multivariable linear regression was performed comparing change in CBF (in all regions of interest [ROIs]) with change in systolic and diastolic BP.

Levine test of equal variances and inspection for normal distribution of residuals were conducted to ensure that ANOVA and regression model assumptions were met. An α value of .05 was used for all tests.

## Results

**Figure 1.**
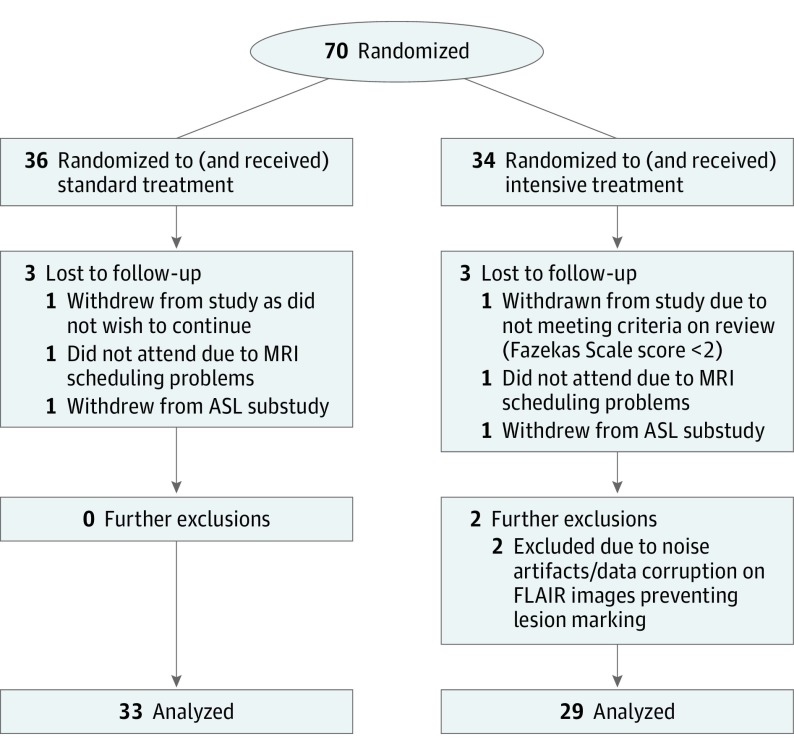
CONSORT Diagram Shown is an overview of the participant flow, sample sizes, and dropout characterization for this analysis. ASL indicates arterial spin labeling; CONSORT, Consolidated Standards of Reporting Trials; FLAIR, fluid-attenuated inversion recovery; and MRI, magnetic resonance imaging.

The PRESERVE trial began recruitment on February 29, 2012. The last patient included was recruited on October 21, 2015. Recruitment across all PRESERVE sites closed on October 30, 2015. Patient flow is shown in Figure 1. Sixty-two participants returned complete, usable data sets (36 at site 1 and 26 at site 2, including 33 in the standard group and 29 in the intensive group). Across this group of 62 participants, the mean age was 69.3 years, and 60% (n = 37) were male; sex distribution within treatment groups was 64% (21 of 33) in the standard group and 55% (16 of 29) in the intensive group. Characteristics of those who dropped out (n = 6) were descriptively similar to those who remained in the study. For those who dropped out, the mean age was 69.4 years, 4 of 6 were male, the mean baseline systolic BP was 152 mm Hg, and the mean baseline Montreal Cognitive Assessment (MoCA) score was 24.50. For those who stayed in the study, the mean age was 69.5 years, 61% (39 of 64) were male, the mean baseline systolic BP was 151 mm Hg, and the mean baseline MoCA was 24.72.

Demographics within treatment groups are listed in Table 1. No variable was significantly different between groups.

**Table 1.** Overview of Demographic and Risk Factor Information Within Each Treatment Group^a^

Variable	Standard (n = 36)	Intensive (n = 34)	*P* Value^b^
Age, mean (SD), y	68.64 (7.92)	70.31 (9.76)	.44
Sex (male:female), No. (%)	24:12 (67:33)	19:15 (56:44)	.35
Race/ethnicity (white:nonwhite), No. (%)	29:7 (81:19)	29:5 (85:15)	.60
Days from stroke to scan, mean (SD)^c^	189.11 (129.64)	202.53 (198.02)	.85
Days between baseline and 3-mo scan (if available), mean (SD)	94.45 (14.86)	97.71 (27.37)	.55
No. of baseline BP medications, mean (SD)	1.53 (1.13)	1.56 (0.96)	.82
Previous stroke (no:yes), No. (%)	30:6 (83:17)	29:5 (85:15)	.82
BP, mean (SD)			
Systolic	150 (10)	153 (12)	.21
Diastolic	84 (10)	87 (13)	.23
MoCA (total), mean (SD)	24.39 (4.02)	25.03 (3.68)	.49
History of depression (no:yes), No. (%)	28:8 (78:22)	29:5 (85:15)	.42
History of cognitive decline (no:yes), No. (%)	23:13 (64:36)	22:12 (65:35)	.94
Diabetes (no:yes), No. (%)	29:7 (81:19)	25:9 (74:26)	.48
Smoker (no:yes), No. (%)			
Current	28:8 (78:22)	31:3 (91:9)	.12
Former	24:11 (69:31)	22:11 (67:33)	.99
Angina (no:yes), No. (%)	32:4 (89:11)	32:2 (94:6)	.44
Myocardial infarction (no:yes), No. (%)	34:2 (94:6)	32:2 (94:6)	.95
Peripheral vascular disease (no:yes), No. (%)^d^	34:1 (97:3)	33:1 (97:3)	.98
Drug-treated hypercholesterolemia (no:yes), No. (%)	4:32 (11:89)	10:24 (29:71)	.06

Abbreviations: BP, blood pressure; MoCA, Montreal Cognitive Assessment.

^a^ Data are presented as means (SDs) for continuous data and as unadjusted ratios (percentages) for frequency data. Some totals do not sum to heading totals because of missing data.

^b^ *P* values (all nonsignificant) compare the treatment groups by groupwise (*t* tests or Mann-Whitney tests) and χ^2^ analyses as appropriate.

^c^ Data are missing for 5 participants (1 standard and 4 intensive).

^d^ Data are missing for 1 participant (standard).

Baseline whole-brain CBF did not differ between the 2 sites. The mean (SD) at site 1 was 30.7 (11.3) mL/min/100 g, and the mean (SD) at site 2 was 33.7 (10.2) mL/min/100 g (*P* = .25).

### BP Measurements in the 2 Treatment Groups

The mean systolic/diastolic BP changed from 150/83 mm Hg at baseline to 141/79 mm Hg at 3 months in the standard group and from 154/88 mm Hg at baseline to 126/75 mm Hg at 3 months in the intensive group, achieving a between-group separation of 15 mm Hg (systolic). Therefore, the mean (SD) systolic/diastolic BP change was 8 (12)/4 (9) mm Hg in the standard group and 27 (17)/13 (13) mm Hg in the intensive group. Change in systolic and diastolic BP was significantly different between treatment groups (*P* < .001 for systolic and *P* = .002 for diastolic) and within treatment groups (standard group *P* = .001 for systolic and *P* = .02 for diastolic; intensive group *P* < .001 for systolic and *P* < .001 for diastolic). At 3 months, 13 of 33 (39%) standard group participants and 18 of 29 (62%) intensive group participants had reached their BP targets.

### Change in CBF Between Treatment Groups

**Figure 2.**
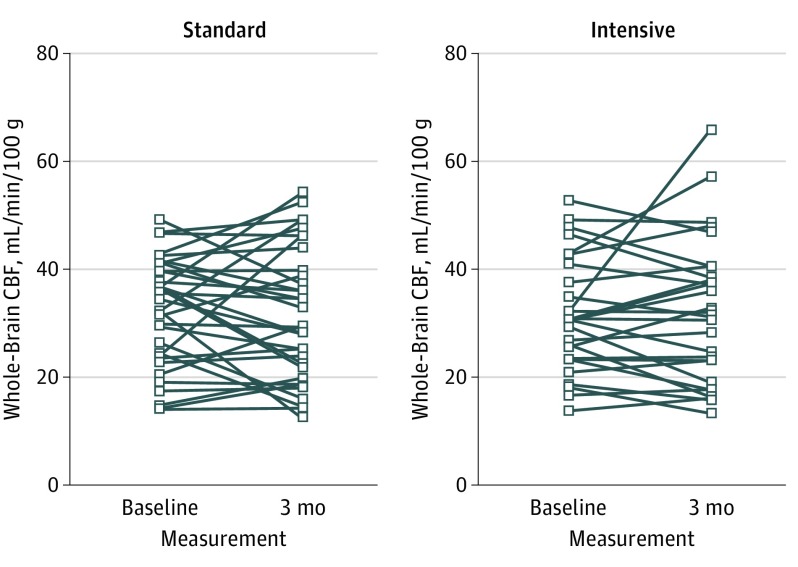
Change in Whole-Brain Cerebral Blood Flow (CBF) The spaghetti plot shows the change for each participant by treatment group.

Cerebral blood flow values across the entire sample were 31.6 mL/min/100 g for whole brain, 45.1 mL/min/100 g for GM, 22.0 mL/min/100 g for all WM, and 22.8 mL/min/100 g for NAWM. Baseline CBF values did not differ between treatment groups in any ROI. Individual participant change in CBF by treatment group is shown in Figure 2.

The primary end point, change in whole-brain CBF, did not differ between the 2 treatment groups: the mean (SD) change was −0.5 (9.4) mL/min/100 g in the standard group vs 0.7 (8.6) mL/min/100 g in the intensive group (partial η^2^, 0.004; 95% CI [based on estimated marginal means], −3.551 to 5.818; *P* = .63). The Bayes factor was 0.217 (ie, these findings were 4.61 times more likely to occur under the null hypothesis, giving moderate support for the null according to conventional interpretation.^29^

There were no differences in CBF change between treatment groups for GM, all WM, or NAWM. Table 2 lists the full results.

**Table 2.** CBF Values at Baseline and 3 Months in the 2 Treatment Groups, With Primary and Initial Secondary End Point ANOVA Results^a^

Variable	Whole-Brain ROI	Gray Matter ROI	All White Matter ROI	Normal-Appearing White Matter ROI
**Standard CBF (n = 33)**				
Baseline/3-mo CBF	32.1 (10.1)/31.7 (12.3)	45.7 (14.3)/46.0 (18.3)	22.3 (7.0)/21.9 (8.3)	23.1 (7.0)/22.7 (8.6)
CBF change	−0.5 (9.4) [*P* = .78]	0.3 (14.2) [*P* = .91]	−0.5 (7.0) [*P* = .71]	−0.5 (7.2) [*P* = .72]
**Intensive CBF (n = 29)**				
Baseline/3-mo CBF	31.1 (10.4)/31.8 (13.2)	44.4 (15.4)/45.7 (18.7)	21.5 (6.6)/21.9 (8.0)	22.5 (7.0)/23.0 (8.6)
CBF change	0.7 (8.6) [*P* = .65]	1.3 (12.2) [*P* = .58]	0.4 (5.6) [*P* = .69]	0.5 (5.8) [*P* = .64]
**ANOVA Findings (Standard vs Intensive Change in CBF)**				
Treatment group findings	*F*_1,58_ = 0.235, partial η^2^ = 0.004, *P* = .63	*F*_1,58_ = 0.049, partial η^2^ = 0.001, *P* = .83	*F*_1,58_ = 0.231, partial η^2^ = 0.004, *P* = .63	*F*_1,58_ = 0.256, partial η^2^ = 0.004, *P* = .62
Estimated model means standard vs intensive difference	Group difference, 1.133; 95% CI, −3.551 to 5.818	Group difference, 0.765; 95% CI, −6.170 to 7.700	Group difference, −0.800; 95% CI, −2.535 to 4.136	Group difference, 0.872; 95% CI, −2.579 to 4.323
Site findings	*F*_1,58_ = 1.319, *P* = .26	*F*_1,58_ = 0.824, *P* = .37	*F*_1,58_ = 0.334, *P* = .57	*F*_1,58_ = 0.255, *P* = .62
Model adjustment *R^2^*	0.019	0.028	0.038	0.036

Abbreviations: ANOVA, analysis of variance; BP, blood pressure; CBF, cerebral blood flow; ROI, region of interest.

^a^ Paired *t* test results that compare change in CBF (milliliters per minute per 100 g) presented as means (SDs) within treatment groups are given below the descriptive values. Univariate ANOVA findings are also shown comparing change in CBF between each treatment group (controlling for site). The 95% CIs are given based on estimated marginal means for the difference in change in CBF between groups. The gray matter CBF model failed the Levine test of equal variances; this analysis was repeated using the Kruskal-Wallis test (without controlling for site) and remained nonsignificant (*P* = .94).

### Secondary Analyses

Further secondary analyses showed no association between change in systolic/diastolic BP and change in CBF: the systolic/diastolic *P* values for ROIs were .73/.88 for whole brain, .67/.87 for GM, .60/.71 for all WM, and .65/.75 for NAWM. There were no differences in CBF change when groups were restricted to standard group participants whose final systolic BP exceeded 130 mm Hg (n = 29) and intensive group participants who reached their target BP (n = 18) (*P* = .89 for whole brain, *P* = .93 for GM, *P* = .95 for all WM, and *P* = .97 for NAWM). Table 3 lists the full results.

**Table 3.** Descriptive and Statistical Values for All Other Secondary End Point Analyses^a^

Variable	Whole-Brain ROI	Gray Matter ROI	All White Matter ROI	Normal-Appearing White Matter ROI
**Restricted Standard CBF (n = 29)**				
Baseline/3-mo CBF	31.0 (10.0)/31.0 (11.8)	43.6 (13.6)/44.9 (17.6)	21.6 (6.9)/21.6 (7.9)	22.4 (7.0)/22.4 (8.3)
CBF change	0.0 (9.3) [*P* = .99]	1.3 (13.7) [*P* = .61]	0.0 (6.6) [*P* = .99]	0.0 (6.8) [*P* = .98]
**Restricted Intensive CBF (n = 18)**				
Baseline/3-mo CBF	31.1 (11.4)/31.2 (15.2)	43.2 (17.0)/43.8 (22.4)	22.2 (7.5)/22.0 (8.8)	23.0 (7.6)/22.9 (9.3)
CBF change	0.1 (10.6) [*P* = .96]	0.5 (14.7) [*P* = .88]	−0.2 (6.5) [*P* = .89]	−0.1 (6.9) [*P* = .95]
**ANOVA Findings (Standard vs Intensive Change in CBF Restricted by Achieved BP Target)**				
Treatment group findings	*F*_1,43_ = 0.021, partial η^2^<0.001, *P* = .89	*F*_1,43_ = 0.008, partial η^2^<0.001, *P* = .93	*F*_1,43_ = 0.004, partial η^2^<0.001, *P* = .95	*F*_1,43_ = 0.001, partial η^2^<0.001, *P* = .97
Estimated model means standard vs intensive difference	Group difference, 0.433; 95% CI, −5.563 to 6.430	Group difference, −0.389; 95% CI, −9.021 to 8.243	Group difference, −0.122; 95% CI, −4.206 to 3.963	Group difference, −0.078; 95% CI, −4.320 to 4.165
Site findings	*F*_1,43_ = 2.264, *P* = .14	*F*_1,43_ = 1.599, *P* = .21	*F*_1,43_ = 0.925, *P* = .34	*F*_1,43_ = 0.765 *P* = .39
Model-adjusted *R^2^*	−0.014	−0.029	−0.044	−0.046
**Change in CBF vs Change in BP Regression Models (All Participants Systolic BP)**				
Change in BP β	−0.046; 95% CI, −0.111 to 0.158 (*P* = .73)	−0.056, 95% CI, −0.155 to 0.241 (*P* = .67)	−0.069, 95% CI, −0.070 to 0.120 (*P* = .60)	−0.060, 95% CI, −0.076 to 0.122 (*P* = .65)
Site β	−0.137 (*P* = .29)	−0.106 (*P* = .42)	−0.062 (*P* = .63)	−0.053 (*P* = .69)
Model-adjusted *R^2^*	−0.011	−0.018	−0.024	−0.027
**Change in CBF vs Change in BP Regression Models (All Participants Diastolic BP)**				
Change in BP β	−0.019; 95% CI, −0.182 to 0.211 (*P* = .88)	0.022; 95% CI, −0.315 to 0.267 (*P* = .87)	−0.050; 95% CI, −0.133 to 0.166 (*P* = .71)	−0.041; 95% CI, −0.122 to 0.168 (*P* = .75)
Site β	−0.138 (*P* = .29)	−0.115 (*P* = .38)	−0.061 (*P* = .64)	−0.052 (*P* = .69)
Model-adjusted *R^2^*	−0.013	−0.021	−0.027	−0.029

Abbreviations: ANOVA, analysis of variance; BP, blood pressure; CBF, cerebral blood flow; ROI, region of interest.

^a^ Cerebral blood flow values (milliliters per minute per 100 g) presented as means (SDs) are given at both time points in treatment groups restricted by achieved BP (where 3-month systolic values were <125 mm Hg for intensive group participants and >130 mm Hg for standard group participants), with paired *t* test results comparing these values given below these. Univariate ANOVA findings are also shown comparing change in CBF between these groups (controlling for site). The 95% CIs are given based on estimated marginal means for the difference in change in CBF between groups. Results from linear regression comparing change in BP vs change in CBF (across all participants) are shown.

### Adverse Events

Mann-Whitney test showed no difference in the number of study drug–related AEs between the 2 groups, with a mean (SD) of 0.21 (0.65) (median, 0) for the standard group and a mean (SD) of 0.32 (0.75) (median, 0) for the intensive group (*P* = .44). Adverse events were reported 29 times, in 17 participants (9 intensive and 8 standard), and were deemed to be related to the study drug in 17 cases, among 10 participants (6 intensive and 4 standard). There was no difference between groups in the number of falls (1 each) or postural related dizziness (2 each). No SAEs were recorded.

## Discussion

In this randomized clinical trial, we investigated the effect of standard vs intensive BP lowering on CBF in patients with severe symptomatic SVD, as manifested by both lacunar stroke and confluent WMHs. Our results showed no difference in change in CBF (of any studied ROI) over the 3-month follow-up period between the 2 treatment groups. Furthermore, we found no excess of AEs in the intensive group, including potentially hypotension-related events. This provides support that intensive BP reduction regimens do not cause hypoperfusion in patients with severe symptomatic SVD.

Intensive BP lowering has been shown to reduce cardiovascular end points in patients without previous cardiovascular disease.^4^ However, there is concern that it may be hazardous in patients with extensive SVD, in whom reduced CBF and impaired cerebral autoregulation have been previously demonstrated.^10^ Therefore, our results are reassuring that intensive treatment can be used in this patient cohort.

These findings complement the Secondary Prevention of Small Subcortical Strokes 3 trial,^12^ which did not measure CBF but applied a similar BP regimen to patients with MRI-confirmed lacunar stroke (but not necessarily confluent WMHs). The intensive treatment was tolerated compared with the standard treatment with regard to outcomes such as SAEs and also showed a trend toward a reduced stroke rate. However, our findings do not support previous work in patients with hypertension without stroke or SVD,^11^ which found that intensive treatment raised CBF via resetting of the cerebral autoregulatory curve. This may be due to patients with SVD having more severe cerebrovascular disease than the elderly participants with hypertension included in that study, meaning that a potential increase either may not be possible because of the extent of their preexisting damage or may take longer to achieve. The PRESERVE study will investigate this last point when the 2-year study is completed.

### Limitations

Our study has limitations. The ASL sequences differed slightly; however, baseline CBF values were not significantly different between sites. Because randomization was stratified across sites, this should not have influenced any analyses. The year the study was planned (2010-2011) also restricted ASL techniques; as such (and to maintain data consistency throughout the study duration), recent advances such as QUIPSS (quantitative imaging of perfusion using a single subtraction) were not used. The lack of a FLAIR scan at the 3-month time point means that new WMHs would be classified as NAWM at follow-up (but NAWM findings did not differ from other ROIs, so this is unlikely to have affected any interpretation). Although we achieved our target treatment group BP difference of 15 mm Hg (systolic), only 62% (18 of 29) of intensive group participants reached their target. This diminishes power for the primary and secondary ANOVAs (by data noise and low numbers, respectively). While the regression analysis (which would be unaffected by this) also supports our conclusion of no connection between BP and CBF change, these considerations, as well as the Bayes factor showing moderate support for the null hypothesis, mean that interpretation should be approached with a degree of caution. Finally, because patients who had a stroke within 3 months were ineligible for the study, our findings are not applicable to acute poststroke treatment.

## Conclusions

While 38% (11 of 29) of intensive group patients failed to reach their target BP, our overall findings are consistent with there being no association between BP lowering and change in CBF (or increased clinical AEs) over a 3-month period compared with standard BP lowering. This suggests that intensive BP lowering does not cause cerebral hypoperfusion in patients with severe SVD.

## Notes

Supplement. **Trial Protocol**
