# Supplementary material for: Effect of Standard vs Intensive Blood Pressure Control on Cerebral Blood Flow in Small Vessel Disease: The PRESERVE Randomized Clinical Trial
Source: JAMA Neurol. 2018 Mar 5;75(6):720–7. doi: 10.1001/jamaneurol.2017.5153 (PMC5885221; doi:10.1001/jamaneurol.2017.5153)
Supplement: Supplement. — Trial Protocol [file jamaneurol-75-720-s001.pdf]

UNIVERSITY OF CAMBRIDGE

Cambridge University Hospitals NHS
NHS Foundation Trust

CLINICAL STUDY PROTOCOL

PRESERVE: How intensively should we treat blood pressure in established cerebral small vessel disease?

Sponsor's R&D Registration Number: A092979
Sponsor's Details: University of Cambridge and Cambridge University Hospitals NHS Foundation Trust (UC and CUH)
EudraCT number: N/A

CHIEF INVESTIGATOR (CI):

Hugh Markus
Addenbrookes Hospital,
Cambridge University Hospitals NHS Foundation Trust,
Hills Road,
Cambridge
CB2 0QQ

Phone: 01223 586 661
Email: hsm32@medschl.cam.ac.uk
Fax: 01223 217 909

SPONSOR REPRESENTATIVE:

Stephen Kelleher, R&D Manager,
Cambridge University Hospitals,
Hills Road,
Cambridge
CB2 0QQ,

Phone: 01223 217418
Fax: 01223 34849
r&denquiries@addenbrookes.nhs.uk

Information in this protocol is confidential and should not be disclosed, other than to those directly involved in the execution or the ethical/regulatory review of the study, without written authorisation Cambridge University Hospitals NHS Foundation Trust and the University of Cambridge or their affiliates.

Signature Page and Statement

The Chief Investigator (CI), UC and CUH have discussed this protocol. The investigators agree to perform the investigations and to abide by this protocol except in the case of medical emergency or where departures from it are mutually agreed in writing.

The Investigator agrees to conduct the trial in compliance with the protocol, GCP and the Data Protection Act (1998), the Trust Information Governance Policy (or other local equivalent), the Research Governance Framework (2005), the Sponsor's SOPs, and other regulatory requirements as appropriate.

This protocol has been written in accordance to the Sponsor's procedure outlined in the Standard Operating Procedure (SOP) identified as: JRODOC001 Protocol Template V1.0.doc

**Chief Investigator**<br>Prof Hugh Markus<br>Professor of Neurology<br>University of Cambridge	Signature	Date
**Sponsor Representative**<br>Stephen Kelleher, R&D Manager<br>Cambridge University Hospitals	Signature	Date

Contents

1 List of abbreviations..
2 Study personnel..
3 Study synopsis..
4 Introduction..
4.1 Background..
5 Study objectives..
5.1 Primary objective..
5.2 Secondary objectives..
6 Trial design..
6.1 Overall design..
7 Eligibility criteria..
7.1 Inclusion criteria..
7.2 Exclusion criteria..
8 Subject/Patient Recruitment process..
9 Study procedures..
9.1 Informed consent..
9.2 Participant Loss of Capacity
9.3 Randomisation procedure..
10 Treatment strategies
11 Study assessments and treatment procedure..
11.1 Screening assessments..
11.2 Baseline assessments..
11.3 Subsequent assessments..
11.4 Summary flow chart of study assessments..
11.5 Laboratory and radiological procedures
11.6 Definition of end of trial
11.7 Drop outs
12 Recording Adverse Events (AEs)..
13 Data management and quality assurance..
13.1 Confidentiality..
13.2 Data collection tool..
13.3 Data handling and analysis..
14 Archiving arrangements..
15 Endpoints..
16 Statistical analysis plan and sample size..
16.1 Sample size calculations..
16.2 Statsitical analysis plan..
16.3 Randomisation..
17 Committees in involved in the trial..
18 Direct access to source data..
19 Ethics and regulatory requirements..
20 Monitoring plan for the trial..
21 Finance and funding..
22 Insurance and indemnity..

23 Publication policy ....
24 Statement of compliance ....
25 References ....

1 List of abbreviations

AE	Adverse Event
AR	Adverse Reaction
ASR	Annual Safety Report
BHS	British Hypertension Society
BP	Blood pressure
CBF	Cerebral blood flow
CI	Chief Investigator
CRF	Case Report Form
CUH	Cambridge University Hospitals
DMC	Data Monitoring Committee
DTI	Diffusion tensor imaging
EudraCT	European Clinical Trials Database
GAfREC	Governance Arrangements for NHS Research Ethics
GCP	Good Clinical Practice
IB	Investigator Brochure
ICF	Informed Consent Form
ISF	Investigator Site File
ISRCTN	International Standard Randomised Controlled Trial Number
Main REC	Main Research Ethics Committee
MMSE	Mini-mental state examination
PI	Principal Investigator
PIS	Participant Information Sheet
QA	Quality Assurance
QC	Quality Control
RCT	Randomised Control Trial
REC	Research Ethics Committee
SAR	Serious Adverse Reaction
SAE	Serious Adverse Event
SDV	Source Document Verification
SOP	Standard Operating Procedure
SSA	Site Specific Assessment
SVD	Cerebral small vessel disease
TMG	Trial Management Group
TSC	Trial Steering Committee
UC	University of Cambridge

2 Study personnel

Principal Investigator (PI): Hugh Markus
Department of Clinical Neurosciences,
R3, Box 83,
Cambridge Biomedical Campus,
Cambridge,
CB2 0QQ

Email: hsm32@medscl.cam.ac.uk

Phone: 01223 586 661

Fax: 01223 217 909

Co-Investigators : Gary A Ford, CBE
Chief Executive Officer, Oxford Academic Health Science Network
Consultant Stroke Physician, Oxford University Hospitals NHS Trust
John Eccles House
Robert Robinson Avenue
Oxford Science Park
Oxford OX4 4GA

Email: Gary.Ford@ouh.nhs.uk

Tom Barrick
Senior Lecturer in Image Analysis
Stroke and Dementia Research Centre
St George's University of London
London, UK

Email: t.barrick@sgul.ac.uk

Jonathan Birns
Consultant in Stroke Medicine, Geriatrics & General Medicine,
Guy's & St Thomas' NHS Foundation Trust
London, UK

Email: jonathan.birns@gstt.nhs.uk

Andrew Blamire
Professor of Magnetic Resonance Physics
Newcastle University, Newcastle, UK

Email: a.m.blamire@newcastle.ac.uk

Robin Morris
Professor of Neuropsychology
King's College, Institute of Psychiatry
London, UK

Email: Robin.Morris@kcl.ac.uk

John O'Brien
Professor of Old Age Psychiatry
Addenbrookes Hospital, Cambridge

Email: john.obrien@medschl.cam.ac.uk

3 Study synopsis

**Full study title:**	How intensively should we treat blood pressure in established cerebral small vessel disease?
**Short study title:**	PRESERVE
**Study R&D number:**	A092979
**Study drug:**	Not applicable
**Chief Investigator:**	Hugh Markus
**Study centres/sites:**	Within the UK Stroke Research Network
**Study duration:**	4 years
**Clinical phase:**	Phase 2
**Primary Objective:**	To determine whether a strategy of intensive, versus standard, treatment of BP in hypertensive individuals with cerebral small vessel disease (SVD) and leukoaraiosis is associated with reduced cognitive decline.
**Secondary Objective:**	1. In a subgroup of the overall RCT to determine whether a strategy of intensive, versus standard, treatment of BP in hypertensive individuals with SVD and leukoaraiosis is associated with brain changes detectable on serial MRI imaging: namely a reduced rate of white matter damage assessed by Diffusion Tensor Imaging, a reduced rate of brain atrophy and an increase in cerebral blood flow (CBF)<br>2. To compare the sensitivity of diffusion tensor MRI and brain atrophy as surrogate markers of white matter damage for therapeutic trials and their relationship to cognitive decline, compared with the conventional MRI marker of T2 white matter lesion volume
**Study population:**	Patients with clinical and radiological features of cerebral small vessel disease
**Methodology:**	Randomised trial of two treatment regimens with primary outcome of change in cognition and secondary outcome of change in MRI parameters
**Study drugs, Dose and Mode of Administration:** Not applicable	
**Duration of Treatment:**	2 years

4 Introduction

4.1 Background

Cerebral small vessel disease (SVD) accounts for about 20% of all stroke (lacunar stroke) and is now recognised to be the major cause of vascular cognitive impairment and dementia (1). Therefore, it presents a major public health problem. It arises from disease in the small perforating arteries supplying the white matter and deep gray matter structures. Radiologically one sees a combination of small discrete lacunar infarcts with or without more diffuse ischaemic changes, best seen on T2-weighted Magnetic Resonance Imaging (MRI) as high signal and referred to as leukoaraiosis (or white matter hyperintensities).

In patients with cognitive impairment due to SVD a characteristic picture of “subcortical” cognitive impairment is seen with impairment of executive function and information processing speed being prominent while episodic memory is preserved.(2,3) Commonly used screening tools for cognitive impairment, such as the Mini Mental State Examination score (MMSE), were designed for the pattern of impairment seen in cortical dementias such as Alzheimer’s disease and are insensitive to the deficits seen in SVD. However, using a cognitive battery tailored to the deficit seen in SVD we have shown (previously and more recently in a Stroke Association project grant) that as many as 50% of patients with lacunar stroke have cognitive deficits.(4,5)

Cognitive deficits are much more common in SVD patients with accompanying leukoaraiosis. Leukoaraiosis in this patient group is related to progressive cognitive impairment and a high risk of developing dementia and disability.(6,7) The recent prospective LADIS study in 639 subjects showed leukoaraiosis specifically contributed to the deterioration in psychomotor speed and executive function which occurred during follow-up.(7) Despite this association between leukoaraiosis and cognition, the correlation between cognition and MRI T2-lesion volume (leukoaraiosis volume) in patients presenting with clinical SVD is weak.(8,9) This may be because T2-high signal does not differentiate between areas of increased water content and structural damage.(10) Using Diffusion Tensor Imaging (DTI) it is possible to better image white matter tract structure, and we have shown that DTI parameters correlate highly significantly with cognitive impairment in this group, and to a greater extent than T2-lesion volume.(8,9,11) This supports a role for white matter tract damage and subsequent cortical-subcortical disconnection in causing cognitive impairment in these patients.

Hypertension is the major risk factor for SVD, being present in up to 80-90% of patients with lacunar stroke and leukoaraiosis.(12) In asymptomatic individuals there is a strong relationship between blood pressure(BP) and leukoaraiosis volume, and treatment of BP reduces leukoaraiosis progression.(13,14) However, the situation is more complex in patients with symptomatic SVD, particularly in those with accompanying leukoaraiosis. In stroke patients as a whole there is strong evidence that treating BP, even in individuals with BP in the "normal" range, reduces recurrent stroke as well as heart and renal disease.(15) However, it has also been suggested that over-zealous treatment of BP in this group with extensive leukoaraiosis could have deleterious consequences.(16) An important mechanism underlying leukoaraiosis is believed to be hypoperfusion in the internal watershed areas at the distal supply of the perforating arteries. We, and others, have confirmed reduced white matter cerebral blood flow (CBF) in SVD,(17,18) and impaired cerebral autoregulation has also been reported.(19) The relative contributions of hypertension and leukoaraiosis to these changes, and the effect of BP lowering on white matter CBF are unclear. Data from the Newcastle group suggest these changes may be reversible. Using MR arterial spin labelling to measure CBF, intensive BP lowering in older people (to target BP <125 mm Hg systolic) increased CBF which was unchanged in a control group treated to a standard BP target (<140 mmHg systolic).(see fig below) Whether similar changes occur in patients with established leukoaraiosis is unknown. (20)

Fig. Data from our study in hypertensive individuals. On the left axial brain slices showing cerebral blood flow (CBF) can be seen. The plot on the right shows baseline CBF values plotted against CBF values at the end of the study. One can see that while baseline values did not differ, follow-up CBF values were higher in the group receiving intensive blood pressure treatment

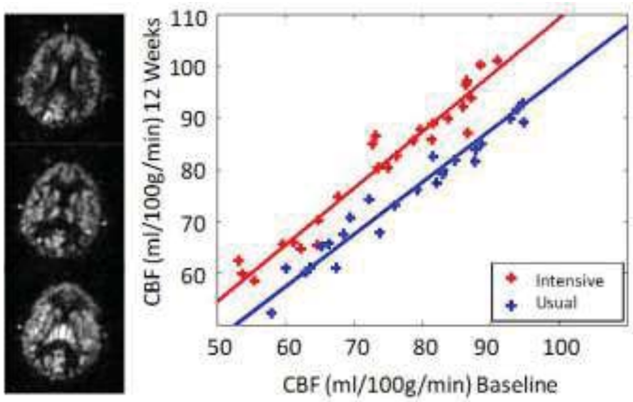

The relationship between hypertensive therapy and cognition is also complex. Prospective longitudinal studies have described an association between raised blood pressure (BP) in midlife and impaired cognition and dementia in later life.(21-23) Studies comparing matched groups of untreated hypertensive and normotensive subjects have demonstrated impairments in some aspects of cognition, even in the absence of clinical vascular disease. (24,25) However the effect of antihypertensive treatment on cognitive function is less clear with differing results in different trials.(26-28) These differences may be partly explained by differing methodology, selective drop

out of those with cognitive decline, and particularly the use of cognitive tests such as the MMSE which are insensitive to the cognitive profile of SVD as outlined above. For example a substudy of the SCOPE trial (29), which overall showed no difference in MMSE between groups (28), found a possible reduction in cognition in the candesartan treated group when more appropriate cognitive tests were used. The above studies have been in unselected individuals with hypertension: the situation is even less clear in patients with SVD and leukoaraiosis. In this group a reduction in BP could be protective due to increasing CBF and/or delaying progression of leukoaraiosis.(16) Conversely it has been proposed that reduced BP could cause a reduction in CBF in individuals with already established SVD and irreversible vessel damage which results in impaired autoregulation, and this could worsen cognition.(16)

Uncertainty about the risk and benefits of BP lowering in this patient group with SVD and leukoaraiosis means that some clinicians treat BP aggressively in this group while others are cautious particularly in older patients.(16) Individual case reports and small series have suggested excessive BP lowering can be hazardous and the optimal target BP remains unclear. There are no randomised controlled trials which have adequately addressed the issue of benefits and risks from different target BP.

We have reviewed the available data (16,30) and identified a number of issues making interpretation difficult:

1. Randomised trials of the effect of BP treatment on cognition have produced conflicting results with no change or trends to improvement in cognition. These studies have generally been underpowered to detect a benefit on cognition, usually because the trial was powered on the basis of vascular outcomes which occur more frequently. Many trials had significant loss to follow up; selective loss of patients at higher risk of cognitive impairment may have masked any treatment effect on cognitive decline (31).
2. There have been no published trials specifically looking at the SVD patient group. Those available have been in normal individuals or individuals with unselected stroke. The pathophysiological picture is quite different in SVD with leuokaraiosis and therefore the research question needs to be asked specifically in this group. The recently funded Prevention Of Decline in Cognition After Stroke Trial (PODCAST) is looking at the important question of whether cardiovascular risk factor treatment in all types of stroke patients prevents subsequent cognitive decline. However it will only have a small proportion of SVD patients with confluent leukoaraiosis and therefore will not be able to answer this question.

3. Most trials have not used appropriate cognitive tests. For example, they have used tests designed for cortical dementias such as Alzheimer's disease. In a recent randomised trial of cholinesterase inhibition in CADASIL (a form of pure SVD and vascular dementia), we could demonstrate no significant treatment effect when using such traditional measures, but we were able to demonstrate a small but significant effect when using more appropriate measures focusing on executive function.(32)
4. MRI can be used as a surrogate outcome to assess treatment but studies to date have used conventional MRI markers of damage. Newer MRI techniques are more sensitive to white matter damage. In a prospective study in SVD patients with leukoaraiosis, we have shown that over a one year period highly significant changes in white matter can be detected using DTI which were not detectable using T2 MRI.(9) These new MRI techniques may allow us to more sensitively and efficiently screen new therapies for SVD, and this would have a huge clinical application in new treatment development.

More detailed pathophysiological studies will also give us information relevant to this clinical question. In particular, it is important to confirm whether or not intensive BP treatment increases CBF in patients with leukoaraiosis. Although we showed reduced CBF in leukoaraiosis patients as a group (17), the exogenous perfusion MRI technique we used was not quantifiable enough to look at changes in response to treatments. Newer endogenous arterial spin labelling (ASL) perfusion techniques are quantifiable and can address this issue. The power of the ASL technique has been shown from the Newcastle studies demonstrating BP lowering increases CBF in older patients with hypertension without SVD (see above). We will apply this technique to SVD with leukoaraiosis to determine the relationship between BP lowering and CBF.

To investigate these questions we will perform this current clinical trial with nested sub-studies. Specifically this will include:

1. A randomised controlled trial (RCT) of intensive versus usual blood pressure lowering treatment with cognition as a primary end point.

2. Nested within the RCT in a subgroup of the overall trial population, a sub-study with progression of white matter damage, assessed using DTI, as the primary endpoint.

3. Nested with the RCT a pathophysiological sub-study determining the effect of intensive blood pressure lowering on cerebral perfusion.

This series of clinical studies will allow us to answer all our research objectives listed above in the most efficient manner.

5 Study objectives

Primary objective

To determine whether a strategy of intensive, versus standard, treatment of BP in hypertensive individuals with SVD and leukoaraiosis is associated with reduced cognitive decline.

Secondary objectives

A. In a subgroup of the overall RCT to determine whether a strategy of intensive, versus standard, treatment of BP in hypertensive individuals with SVD and leukoaraiosis is associated with brain changes detectable on serial MRI imaging:
   - A reduced rate of white matter damage assessed by Diffusion Tensor Imaging
   - A reduced rate of brain atrophy (global, or grey or white matter)
   - An increase in CBF

B. To compare the sensitivity of diffusion tensor MRI and brain atrophy as surrogate markers of white matter damage for therapeutic trials and their relationship to cognitive decline, compared with the conventional MRI marker of T2 white matter lesion volume.

6 Trial design

6.1 Overall design

This is a randomised trial of two treatment strategies (intensive versus standard) for lowering blood pressure in patients with SVD and radiological leukoaraiosis.

Within this overall study there will be two nested sub-studies which some, but not all, patients will also enter:

1. DTI MRI substudy
2. Perfusion MRI study

Undertaking such a treatment trial double blind would be very difficult and previous trials of BP lowering intensity (*e.g.* HOT) have not used double blind treatment regimens. To avoid bias in outcome assessment there will be blinded assessment of the following outcomes:

- Cognitive assessment scores
- MRI data
- Clinical end points

7 Eligibility criteria

7.1 Inclusion criteria

1. Clinical evidence of cerebral small vessel disease, characterised by either:

   - Lacunar stroke syndrome with symptoms lasting >24 hours

   OR

   - Transient ischaemic attack lasting < 24 hours with limb weakness, hemisensory loss or dysarthria AND with MR DWI imaging performed acutely showing lacunar infarction, or if MRI is not performed acutely (>2 weeks after TIA) with a lacunar infarction in an anatomically appropriate position on MRI

   OR

   - Vascular cognitive impairment with MRI showing no evidence of hippocampal atrophy (34)

2. MRI evidence of lacunar infarct(s) (<=1.5cm maximum diameter) and confluent leukoaraiosis (defined on Fazekas scale as >=grade 2) (33).

3. Systolic BP > 140 mmHg and taking no more than two BP lowering drugs

   OR

   Systolic BP between 125 and 140 mmHg with past history of hypertension and on at least one and not more three BP lowering drugs.

4. Age >40 years

5. No diagnosis of dementia on DSM IV criteria

6. Able and willing to consent

7. Expected life expectancy > 2 years

8. Able to perform study cognitive assessments

Patients will be studied >3 months after most recent stroke to avoid confounding by effects of recovery from acute stroke on cognition.

7.2 Exclusion criteria

1. Unable or unwilling to consent
2. Women of childbearing potential
3. Diagnosis of dementia on DSM IV criteria
4. Life expectancy less than 2 years
5. Symptomatic postural hypotension
6. Known single gene disorder causing small vessel disease (eg CADASIL)
7. Cortical infarction (>2 cm maximum diameter)
8. Symptomatic carotid stenosis or vertebral stenosis >50% as measured on NASCET criteria

7.3 Vascular Cognitive Impairment

To be entered into the study with vascular cognitive impairment, the patient must meet each of the following criteria:

1. Cognitive concern reflecting a change in cognition reported by patient or informant or clinician (i.e., historical or observed evidence of decline over time).
2. Cerebrovascular disease thought to underpin the cognitive impairment (i.e. other causes such as medication, metabolic, infective, endocrine or psychiatric disorders, or underlying Alzheimer's disease, are not suspected).
3. MoCA score of 25 or less
4. MRI showing no evidence of hippocampal atrophy

8 Subject/Patient Recruitment process

Patient recruitment at a site will only commence once the trial team has ensured that the following approval/essential documents are in place:

1. The main REC approval,
2. Final sponsorship and host site approval,

3. Sponsor has conducted the trial initiation procedure.

All sites participating in the trial will also be asked to provide a copy of the following:

1. Host site (R&D approval)
2. Signed Delegation of Duties and Responsibilities Logs

Patient will be recruited from

1. Stroke, Neurology, Health care of the Elderly, Old Age Psychiatry, and Medicine Stroke Units and in patient services
2. Stroke, Neurology, Health care of the Elderly, Old Age Psychiatry, and Medicine Out-patient clinics
3. Review of stroke and other similar disease registers
4. Review of discharge summaries and radiology reports

9 Study procedures

9.1 Informed consent

All patients will give informed consent. Informed consent must be obtained before any trial-related procedures are undertaken.

Informed consent will be taken by medical staff at SpR level or above or Senior Research nursing staff (Band 6 and above).

Patient will be given up to 7 days to decide whether they wish to take part.

9.2 Participant Loss of Capacity

Because cognitive dementia is common among patients with cerebral small vessel disease, it is possible that individual participants may lose capacity to consent over the course of the study. At the point of recruitment, it is specified in the inclusion criteria that all participants must be able to provide consent to join the study. If, during the course of the study, there are any concerns about a participant's capacity to consent (using the Mental Capacity Act (2005) as a guide), a consultee will be approached to provide advice on the participant's continued participation. We will ask the participant to identify a consultee, usually a partner or family member or carer, at the baseline visit

9.3 Randomisation procedure

Patients will be randomised to Usual or Intensive blood pressure lowering. Randomisation will be in the ratio 1:1 and performed via an online randomisation system, available 24 hours, based at the Mental Health & Neuroscience Clinical Trials Unit (MH&N CTU) at the Institute of Psychiatry. Randomisation will be stratified by centre.

10 Treatment strategies

Patients will be randomised between two treatment strategies:

1. Intensive BP lowering: aiming for a systolic BP of <125mmHg
2. Usual blood pressure lowering: aiming for a systolic BP of 130-140mmHg, as recommended by current guidelines

This is a trial comparing two strategies for lowering blood pressure and not of specific blood pressure drugs. We are not ascertaining, verifying or comparing efficacy of medicines, only different BP targets achieved by pharmacological and non-pharmacological means. BP lowering management will be the responsibility of the local PI at each site. Sites will be provided with recommended treatment algorithms for intensive and usual BP lowering protocols consistent with the British Hypertension Society (BHS)/NICE guidance on drug treatment of hypertension. These have been developed by the Newcastle group as part of a Biomedical Research Centre Programme study. Using these algorithms, in a group of older hypertensives BP was reduced from 149/87 to 123/70 mm Hg in the Intensively treated group compared to 155/84 to 140/79 mm Hg in the usual group. (Ford, unpublished observations)

Both groups will be given home blood pressure monitors and asked to perform daily blood pressure readings for at least three days prior to each pre-arranged telephone follow-up. On each occasion they will take a reading in the seated position always from the same arm (left unless specified).

1. The intensive BP lowering group will have BP lowering treatment increased at baseline assessment and be reviewed by telephone at two weekly intervals. If average BP at any follow-up is >125 mmHg treatment will be increased until target systolic BP of <125 mm Hg is achieved (average of 2nd and 3rd of three seated BP readings), or symptoms of hypotension prevent treatment being intensified. If dose of an existing drug is instituted this can be done over the telephone but if a new agent is required the patient will attend for the prescription.

2. The usual BP lowering group will have treatment unchanged at study entry. They will be contacted for two weekly intervals for the first month and then seen for regular follow-up as outlined blow. At follow-up if average systolic BP is above 140 mmHg treatment will be increased until target systolic BP of <140mmHg or symptoms of hypotension prevent treatment being intensified.

Note: Patients taking part in the perfusion MRI study will not have their treatment altered until they have had their baseline MRI scan.

The NICE/British Hypertension Society guideline based algorithm will act as a guide to treatment but all treatment decisions will be made by the local principal investigator. In both treatment groups treatment will be changed if the patient experiences adverse effects considered by the local PI to be related to the BP lowering treatment.

11 Study Assessments

11.1 Screening assessments

At a screening assessment potential study participants will be reviewed with the following to ensure they meet the inclusion criteria and do not have exclusion criteria and the researcher will fill in a screening checklist. Assessments will include:

1. Review of medical records and clinical history to confirm clinical diagnosis consistent with cerebral small vessel disease

2. Review of MRI brain imaging to confirm presence of lacunar infarction and confluent leukoaraiosis (>= Fazekas grade 2)- in conjunction with leukoaraiosis grading package

3. Baseline medical examination including BP. Study BP measurements will be taken as the mean of the last two of three reading taken in the sitting position after 5 minutes rest.

4. Review of current medication including number and doses of antihypertensive agents to ensure appropriate prior to study entry.

11.2 Baseline assessments

11.2.1 Clinical assessment

1. Clinical history
2. Neurological and cardiovascular examination
3. Modified Rankin Score
4. Measurement of seated and standing BP
5. Recording of current medication

11.2.2 Assessment of cognition

The primary outcome will be changes in a composite cognitive score with cognition assessed by a battery of tests known to be sensitive to impairments in attention, information processing and executive function due to subcortical white matter disease (1). This will yield a composite score comprising data from the following tests. Times each test takes are shown in brackets.

A) The Trail Making Tests (TMTs) (35), using the sequencing (TMT-A) and dual conceptual tracking (TMT-B) subtests; (5 minutes)
B) Digit symbol Coding a measure of processing speed from the WAIS IV (36) (3 minutes); and
C) Controlled Oral Word Association (FAS) (36) (4 minutes) to assess phonemic verbal fluency and the Category Fluency test (Animals) (36) (2 minutes) to asses semantic verbal fluency

Secondary outcome measures will include the following:

A) The Montreal Cognitive Assessment (MOCA) (37), a brief freely available global measure of cognition designed for patients with Mild Cognitive impairment (MCI) but more relatively more heavily weighted to subcortical cognitive dysfunction compared with the MMSE; (7 minutes)
B) The Rey-Auditory Verbal Learning Test (RAVLT) (38) will assess learning and memory, this test is sensitive to the memory disorder associated with Alzheimer's disease (AD) type pathology (13 minutes)

In addition premorbid intellectual functioning at baseline will be assessed using the National Adult Reading Test - Restandardised (NART-R) (39) (5 minutes).

11.2.3 Other asssessments

a) Disability assessment for dementia (DADS) (40) which assesses the patients ability to do basic Activities of Daily Living (ADL) and instrumental ADL (IADL). This assessment is administered to a carer or other informant who knows the patient sufficiently well to respond to the questions accurately.
b) The Stroke Specific Quality of Life assessment (SSQoL),(41) which we have shown detects a reduction in QoL in patients with lacunar stroke and leukoaraiosis, compared with age matched controls.(42) and a more generic measure, the EUROQOL(http://www.euroqol.org/home.html), to capture areas not well covered by the SSQoL, such as those relating to cognition.

After consent clinical MRI scans will be sent centrally (or to designated satellite reading centres) for review to confirm radiological eligibility.

MRI will only be performed specifically as part of the study at baseline for those in the MRI substudies- details of these scans are outlined in section 11.5.2.

At baseline the contact details of a partner/relative or other close informant will also be taken to allow information to be collected if the participant loses capacity during the study.

11.3 Subsequent assessments

All subjects will be seen at 1, 3, 6, 12, 18 and 24 months (measured from the baseline visit) for a clinical assessment and monitoring of BP. Cognitive assessments will be performed at 1 year and 2 years except for the MOCA which will also be performed at 3 months. When a cognitive assessment is required at the same time, both will be carried out on the same visit.

Clinical assessment at 1, 3, 6, 12, 18 and 24 months

1. Documentation of current medication
2. Measurement of sitting and standing blood pressure; latter determined after last of 3 seated readings.
3. Review of any adverse effects and specific enquiry about postural related dizziness and falls
4. Clinician review to determine if BP remains appropriately controlled for standard or intensive BP targets and if not, BP lowering drug therapy reviewed and altered as appropriate.

Cognition assessment at 12 and 24 months

1. The Trail Making Tests (TMTs) sequencing (TMT-A) and dual conceptual tracking (TMT-B) subtests
2. Digit symbol Coding a measure of processing speed from the WAIS IV;
3. Phonemic (COWAT; FAS) and semantic verbal fluency (Animals).
4. The Montreal Cognitive Assessment (MOCA) – also performed at 3 months
5. The Rey-Auditory Verbal Learning Test (RAVLT)
6. Disability assessment for dementia (DADS)
7. The Stroke Specific Quality of Life assessment (SSQoL)
8. EUROQOL

MRI will only be performed specifically as part of the study at follow-up for those in the MRI substudies- details of these scans are outlined in section 11.6.

11.4 Summary flow table of study assessments

See Appendix 1 and 2

11.5 Laboratory and radiological procedures

11.5.1 Laboratory procedures

Blood will be taken at baseline for both DNA extraction and storage of serum. Genotyping of polymorphisms discovered as part of ongoing genome wide association studies will be performed.

Blood will be taken by venupuncture into tubes for serum and into EDTA tubes for DNA. Serum samples will be centrifuged and serum separated and pipetted into a storage tube and both extracted serum and EDTA will be stored in a freezer at ≤ -70°C in the local centre until transferred to CUH. Extracted DNA samples may be sent for additional genotyping to other centres in the UK or Europe for specific analyses as part of future collaborative projects.

11.5.2 Radiology or any other procedure(s)

Only patients who have undergone an MRI brain scan prior to screening can be assessed for suitability for the study. It is anticipated that patients will have already had an MRI scan performed for clinical management purposes. Patients who have a MRI scan planned can be screened for eligibility prior to this being performed. All MRI used at screening to confirm eligibility must have been performed within 2 years of randomisation and the patient must have suffered no new stroke with residual disability since the scan was performed.

Only patients within the MRI substudies will have MRI scans as part of the study. All patients recruited to the MRI substudies will be part of the larger RCT. Subjects can be in either or both of the MRI substudies. All of these additional MRI scans will be performed using clinical MRI scanners at field strengths of 1.5 or 3T and do not involve exposure to radiation. MRI does not involve radiation and there will be no contrast administration.

11.5.2.1 DTI-MRI sub-study

A standardised MRI protocol will be performed at baseline and after 2 years.

This will include:

- high resolution 3D T1-weighted images (for brain volume) ~4.5 minutes
- T2-weighted gradient echo (GE) images (for identification of microbleeds) ~7 minutes
- FLAIR (for computation of lesion volume) ~4.5 minutes
- DTI (for white matter structural analysis). DTI provides quantitative measures (fractional anisotropy and diffusivity (including axial, radial and mean diffusivity) which can be compared both longitudinally and across sites. High angular resolution diffusion-weighted images will be acquired using a pulsed gradient spin echo planar imaging (EPI) sequence in approximately 11 minutes.

11.5.2.2 Perfusion sub-study

Subjects in this substudy will have the standard MRI protocol above and in addition will have cerebral blood flow (CBF) / perfusion studies CBF MRI will be performed at baseline, 3 months, and at 2 years.

The CBF MRI will take place in two sites (Newcastle and St George's) only. These sites have similar 3 Tesla Phillips MR systems. Randomisation will be stratified by study centre/scanner (St George's v Newcastle) reducing bias.

The arterial spin labelling (ASL) technique we will use has been implemented at Newcastle in studies on hypertensive individuals and identical protocols will be used in each centre. CBF is measured using an ASL sequence with Gradient Echo (GE) Echo Planar. 12 contiguous transverse slices are positioned parallel to the anterior commissure – posterior commissure line with the centre of the sampled volume passing through the most anterior part of the corpus callosum. To avoid contrast reduction due to large transit zone in ASL, data are acquired in 3 separate but contiguous segments with identical FLAIR protocols each containing 4 contiguous slices. The ASL images are motion corrected using Automated Image Registration (AIR 5.2.5), and then split into tag and control image sets. Perfusion weighted images (dM) are generated by taking the difference between the 2 sets, and magnitude images (M) obtained by averaging the 2 sets.

Each ASL scan will take about 20 minutes

11.6 Definition of the End of Trial

This is the Last Patient Last Visit (LPLV) (*i.e.* telephone call, home visit, hospital visit).

11.7 Drop outs

If subjects discontinue for any reason, if possible an assessment including cognitive assessment will be performed just prior to study exit

12 Recording Adverse Events (AEs)

A record of adverse events will be recorded and the relationship to treatment assessed and forwarded to the study co-ordinating centre

A record of adverse events will be collected at each follow-up visit.

Specifically we will ask at each visit about

- Falls
- Dizzyness/postural instability

13 Data management and quality assurance

13.1 Confidentiality

All data will be handled in accordance with the Data Protection Act 1998.

The Case Report Forms (CRFs) will not bear the subject's name or other personal identifiable data. The subject's initials, Date of Birth (DOB) and trial Identification Number (ID), will be used for identification.

13.2 Data collection tool

All on case report forms, data will be entered legibly in black ink with a ball-point pen. If the Investigator makes an error, it will be crossed through with a single line in such a way to ensure that the original entry can still be read. The correct entry will then be clearly inserted. The amendment will be initialled and dated by the person making the correction immediately. Overwriting or use of correction fluid will not be permitted.

It is the Investigator's responsibility to ensure the accuracy of all data entered and recorded in the CRFs. The Delegation of Responsibilities Log will identify all trial personnel responsible for data collection, entry, handling and managing the database.

13.3 Data handling and analysis

Data will be entered onto the online InferMed MACRO data entry system, hosted at the Mental Health and Neurosciences Clinical Trials Unit. The system is compliant with GCP, with a full audit trail and formal database lock functionality.

Data will also be stored on a two level password protected database in the Stroke and Dementia Research Centre at St George's University of London. The database will be backed up daily on the St George's server. In time, data will be moved to a secure area at Cambridge University Hospitals NHS Foundation Trust. All electronic transfer will be done in accordance with the Data Protection Act 1998.

The trial will be coordinated from the Neurology Unit at CUH, but managed by a full time study manager based at the Stroke and Dementia Research Centre, St George's University of London. In time, study management will transfer to CUH completely. The Study Manager will, in either case, be supervised by and report to the trial steering committee. They will also work closely with the MHClinical Trials Unit, which will assist in study monitoring. An independent Data Monitoring Committee will monitor trial progress.

Data entry quality will be checked by a random check on CRFs and their corresponding database entry

14 Archiving arrangements

14.1 Site Archiving

The trial documents (including the site File (SF), Informed Consent Forms along with the CRFs will be kept at sites for a minimum of five years, in line with local hospital protocol.

14.2 Trial Management Archiving

The Trial Master File will be stored in locked offices within the CUH site. The Chief Investigator is responsible for the secure archiving of trial documents which will be archived at CUH. The trial database will also be kept electronically on the UC computer network, for a minimum of five years.

15 Endpoints

15.1 Main study

15.1.1 Primary endpoints

Composite cognitive score

15.1.2 Secondary endpoints

a. Specific cognitive tests

1. The Trail Making Tests (TMTs)
2. Digit symbol Coding
3. Verbal Fluency.
4. The Montreal Cognitive Assessment (MOCA) score
5. The Rey-Auditory Verbal Learning Test (RAVLT)

b. Disability measures

1. Disability assessment for dementia (DADS)
2. Activities of Daily Living (ADL)
3. Instrumental ADL (IADL)

c. Quality of Life

1. Stroke specific QOL
2. EUROQOL

d. Blood pressure: systolic, diastolic and mean

e. Adverse events

15.2 Structural DTI MRI sub-study

15.2.1 Primary endpoints

DTI white matter ultrastructure measured by MD and FA

15.2.2 Secondary endpoints

1. Brain atrophy
2. White matter lesion volume measured on T2/FLAIR

15.3 Perfusion MRI sub-study

15.3.1 Primary endpoint

Cerebral blood flow

16.0 Statistical analysis and sample size

16.1 Sample size calculation

16.1.1 Main study with cognitive endpoint

Sample sizes are based on our pilot study in 25 individuals (30), and also informed by a review of data from our treatment trial in the genetic form of small vessel disease CADASIL(32), experience from the Newcastle SCOPE and PRoFESS COG cognition studies, and a review of the literature (16). Our endpoint in the current study will be a composite cognitive score. In our pilot study we used a similar executive function composite score based on a choice reaction time test, a verbal fluency test, a digit span test, and the difference between the TMT-B and TMT-A times, and the difference between the TMT-B and trail testing motor speed times. Cognitive testing in multicentre studies has higher variance than in single centre studies and therefore we have increased the sigma used below (standard deviation of the outcome measurement in the control group) by 30% from those values we obtained from our single centre pilot study. Calculations were performed in PS Power and Sample Size Calculations Version 3.0, January 2009 (http://biostat.mc.vanderbilt.edu/PowerSampleSize), with a two-sided significance level of 5% and power of 90%. We have estimated final numbers after assuming an attrition rate of 10%.

		Power 0.8			Power 0.9		
delta	sigma	M	N	N-A	M	N	N-A
0.065	0.196	144	288	316	192	384	422

delta = difference between control and intervention groups in the feasibility study

sigma = standard deviation of the outcome measurement in the control group

M = number of subjects required in each group

n = total number of subjects required in study

N-A = total number of subjects after accounting for attrition

16.1.2 DTI-MRI substudy

We will include 180 subjects (90 in each arm) in this substudy; sample sizes are based on FA values from our DTI longitudinal study. With p<0.05, and power of 0.9, and with SD in control group of 6.0 x 10-3 and difference between 2 interventions of 3.1x10-3 we require 80 in each group ie 160 total, which we have increased by 12.5 % (a figure based on our SCANS prospective MRI study) to 180 to account for attrition.

16.1.3 Perfusion substudy

Global CBF, and white and grey matter CBF will be determined. We will determine whether there are significant differences in change in CBF between the two groups. In our recent COGFAST study we used our ASL method in elderly controls (n=30, mean age 83 +/- 2.4 years) and measured white and grey matter. Values were: grey matter 41.7 +/- 10.3 ml/100g/min, white matter WM: 23.8 +/- 5.3 ml/100g/min. Based on this WM data and with our planned group sizes of 30 per group (60 total) we will have a power of 0.9 to detect a reduction in WM CBF 24% at p=0.01.

16.2 Statistical analysis plan

16.2.1 Primary endpoint analysis

To avoid bias due to multiple testing of the large number of neuropsychological tests a composite executive function score will be calculated by averaging z scores (calculated using the DKEFS normative data) for the Trail Making, Digit Symbol Coding and Verbal Fluency tests. For Trail Making adjusted outcome scores will be the difference between the TMT-B and TMT-A times and the difference between the TMT-B and motor speed. Data will be analysed on an intention to treat basis.

The primary analysis will be change between baseline and year 2.

The primary analysis will be intention to treat after patients excluded after review of the screening scan for not having small vessel disease/lacunar infarction have been excluded

A per protocol analysis will also be performed.

16.2.2 Secondary endpoint analysis

The secondary endpoint analysis will be intention to treat after patients excluded after review of the screening scan for not having small vessel disease/lacunar infarction have been excluded

A per protocol analysis will also be performed.

16.3 Randomisation

There may be differences between sites in MRI system characteristics and for this reason randomisation to the MRI substudy is stratified by site.

17. Committees in involved in the trial

1. Trial Management Group (TMG) – This will be responsible for day-to-day management of the trial and will comprise the CI, the trial manager, and study clinical research fellows and neuropsychologist. They will co-ordinate regular teleconferences (eg monthly) with co-ordinating staff from St. George's University of London, University of Cambridge, Oxford University and Newcastle University. The role of the group is to monitor all aspects of the conduct and progress of the trial, ensure that the protocol is adhered to and take appropriate action to safeguard participants and the quality of the trial itself.

2. Trial Steering Committee (TSC) - This will provides overall supervision of the trial and ensures that it is being conducted in accordance with the principles of GCP and the relevant regulations.

3. Independent Data Monitoring Committee (IDMC) – This will regularly monitor trial progress assess whether there are any safety issues that should be brought to participants' attention or any reasons for the trial not to continue.

18. Direct access to source data

The Investigator(s)/institution(s) will permit trial-related monitoring, audits, REC review, and regulatory inspection(s), providing direct access to source data/documents. Trial participants are informed of this during the informed consent discussion. Participants will consent to provide access to their medical notes.

19. Ethics and regulatory requirements

The Sponsor will ensure that the trial protocol, Patient Information Sheet (PIS), Informed Consent Form (ICF), GP letter and submitted supporting documents have been approved by a main Research Ethics Committee (REC), prior to any patient recruitment taking place. The protocol and all agreed substantial protocol amendments, will be documented and submitted for ethical and regulatory approval prior to implementation.

Before site(s) can enrol patients into the trial, the Principal Investigator must apply for Site Specific Assessment from the Trust Research & Development (R&D) and be granted written NHS R&D approval. It is the responsibility of the Principal Investigator at each site to ensure that all subsequent amendments gain the necessary approval. This does not affect the individual clinician's responsibility to take immediate action if thought necessary to protect the health and interest of individual patients (see section 12 for details of reporting procedures/requirements).

Within 90 days after the end of the trial, the CI and Sponsor will ensure that the main REC are notified that the trial has finished. If the trial is terminated prematurely, those reports will be made within 15 days after the end of the trial.

The CI will supply a summary report of the clinical trial to the main REC within one year after the end of the trial.

20. Monitoring plan for the trial

The trial will be monitored according to the monitoring plan agreed and written by the Sponsor, based on the internal risk assessment procedure.

21. Finance and funding

The study is funded by a Stroke Association/British Heart Foundation Programme Grant.

22. Insurance and indemnity

NHS bodies are liable for clinical negligence and other negligent harm to individuals covered by their duty of care. NHS Institutions employing researchers are liable for negligent harm caused by the design of studies they initiate. The provision of such indemnity for negligent harm should be stated to the participant.

23. Publication policy

Results will be published in peer reviewed journals and presented at conferences. Publications policy will be decided by the steering committee.

24. Statement of compliance

The trial will be conducted in compliance with the protocol, Sponsor's Standard Operating Procedures (SOPs), GCP and the applicable regulatory requirement(s).

The study conduct shall comply with all relevant laws of the EU if directly applicable or of direct effect and all relevant laws and statutes of the UK country in which the study site is located including but not limited to, the Human Rights Act 1998, the Data Protection Act 1998, the Medicines Act 1968, the Medicines for Human Use (Clinical Trial) Regulations 2004, and with all relevant guidance relating to medicines and clinical studies from time to time in force including, but not limited to, the ICH GCP, the World Medical Association Declaration of Helsinki entitled 'Ethical Principles for Medical Research Involving Human Subjects' (2008 Version), the NHS Research Governance Framework for Health and Social Care (Version 2, April 2005).

This study will be conducted in compliance with the protocol approved by the REC and according to GCP standards. No deviation from the protocol will be implemented without the prior review and approval of the Sponsor and REC except where it may be necessary to eliminate an immediate hazard to a research subject. In such case, the deviation will be reported to the Sponsor and REC as soon as possible.

25. References

1. Román GC, Erkinjuntti T, Wallin A, Pantoni L, Chui HC. Subcortical ischaemic vascular dementia. Lancet Neurol. 2002;1:426-36.

2 O'Brien JT, Erkinjuntti T, Reisberg B, Roman G, Sawada T, Pantoni L, Bowler JV, Ballard C, DeCarli C, Gorelick PB, Rockwood K, Burns A, Gauthier S, DeKosky ST. Vascular cognitive impairment. Lancet Neurol. 2003;2:89-98.

3. Graham NL, Emery T, Hodges JR. Distinctive cognitive profiles in Alzheimer's disease and subcortical vascular dementia. J Neurol Neurosurg Psychiatry 2004;75:61–71

4. O'Sullivan M, Morris RG, Markus HS. Brief Cognitive Assessment for Patients with Cerebral Small Vessel Disease. J Neurol Neurosurg Psychiatry 2005;76:1140-5.

5. Brookes RL, Hannesdottir K, Lawrence R, Morris RG, Markus HS. The Brief Memory and Executive Test (BMET): A rapid cognitive screening test for vascular cognitive impairment.(submitted)

6. Jokinen H, Kalska H, Ylikoski R, Madureira S, Verdelho A, van der Flier WM, Scheltens P, Barkhof F, Visser MC, Fazekas F, Schmidt R, O'Brien J, Waldemar G, Wallin A, Chabriat H, Pantoni L, Inzitari D, Erkinjuntti T; LADIS group. Longitudinal cognitive decline in subcortical ischemic vascular disease--the LADIS Study. Cerebrovasc Dis. 2009;27:384-91.

7. Inzitari D, Pracucci G, Poggesi A, Carlucci G, Barkhof F, Chabriat H, Erkinjuntti T, Fazekas F, Ferro JM, Hennerici M, Langhorne P, O'Brien J, Scheltens P, Visser MC, Wahlund LO, Waldemar G, Wallin A, Pantoni L; LADIS Study Group. Changes in white matter as determinant of global functional decline in older independent outpatients: three year follow-up of LADIS (leukoaraiosis and disability) study cohort. BMJ. 2009 Jul 6;339:b2477. doi: 10.1136/bmj.b2477

8. O'Sullivan M, Morris RG, Huckstep B, Jones DK, Williams SCR, Markus HS. Diffusion tensor MRI correlates with executive dysfunction in patients with ischaemic leukoaraiosis. J Neurolog Neurosurg Psychiatry 2004;75:441-7.

9. Nitkunan A, Barrick TR, Charlton RA, Clark CA, Markus HS. Mutlimodal MRI in Cerebral Small Vessel Disease; its relationship with cognition and sensitivity to change over time. Stroke. 2008;39:1999-2005

10. Awad I, Johnson P, Spetzler R, Hodak J. Incidental subcortical lesions identified on magnetic resonance imaging in the elderly. II. Postmortem pathological correlations. Stroke 1986;17:1090-7.

11. O'Sullivan M, Summer P, Jones D, Jarosz J, Williams S, Markus H. Normal appearing white matter in ischaemic leukoaraiosis: A diffusion tensor MRI study. Neurology 2001;57:2307-10.

12. Khan U, Porteous L, Hassan A, Markus H. Risk factor profile of cerebral small vessel disease and its subtypes. J Neurol Neurosurg Psychiatry. 2007;78:702-6

13. Breteler MM, van Swieten JC, Bots ML, Grobbee DE, Claus JJ, van den Hout JH, et al. Cerebral white matter lesions, vascular risk factors, and cognitive function in a population-based study: the Rotterdam Study. Neurology 1994;44:1246-52.

14. Dufouil C, Chalmers C, Coskun O, Besancon V, Bousser M-G, Guillon P, et al. Effects of blood pressure lowering on cerebral white matter hyperintensities in patients with stroke. The PROGRESS (Perindopril protection against recurrent stroke study) magnetic resonance imaging substudy. Circulation. 2005; 112: 1644-1650

15. PROGRESS Collaborative Group. Randomised trial of a perindopril-based blood pressure-lowering regimen among 6105 individuals with previous stroke or transient ischaemic attack. Lancet 2001; 358: 1033-1041

16. Birns J, Markus HS, Kalra L. Blood pressure reduction for vascular risk – is there a price to be paid? Stroke 2005 ;36:1308-13

17. Markus HS, Lythgoe DJ, Ostegaard L, O'Sullivan M, Williams SCR. Reduced white matter CBF in ischaemic leukoaraiosis demonstrated using quantitative exogenous contrast based perfusion MRI. J Neurol Psychiat Neurosurg 2000; 69: 48-53.
18. Yao H, Sadoshima S, Kuwabara Y, Ichiya Y, Fujishima M. Cerebral blood flow and oxygen metabolism in patients with vascular dementia of the Binswanger type. Stroke 1990;21:1694-1699.
19. Birns J, Jarosz J, Markus HS, Kalra L. Cerebrovascular reactivity and dynamic autoregulation in ischaemic subcortical white matter disease. J Neurol Neurosurg Psychiatry. 2009;80:1093-8.
20. Intensive blood pressure lowering increases cerebral blood flow in older subjects with hypertension. He J, Tryambake D, Firbank MJ, O'Brien JT, Ford GA, Blamire AM. 2010. Proc ISMRM 18, p 2222 (abstract)
21. Elias MF, Wolf PA, D'Agostino RB, Cobb J, White LR. Untreated blood pressure level is inversely related to cognitive functioning: The Framingham Study. Am J Epidemiol 1993;138:353–364.
22. Launer LJ, Masaki K, Petrovitch H, Foley D, Havlik RJ. The association between midlife blood pressure levels and late-life cognitive function: The Honolulu-Asia Aging Study. JAMA 1995;274:1846–1851.
23. Skoog I, Lernfelt B, Landahl S, et al. 15-year longitudinal study of blood pressure and dementia. Lancet 1996; 347:1141–1145.
24. Harrington F, Saxby BK, McKeith IG, Wesnes K, Ford GA. Cognitive performance in hypertensive and normotensive older subjects. Hypertension 2000;36:1079–1082.
25. Kalra L, Jackson SH, Swift CG. Psychomotor performance in elderly hypertensive patients. J Hum Hypertens1993;7:279–284.
26. Applegate WB, Pressel S, Wittes J, et al. Impact of the treatment of isolated systolic hypertension on behavioral variables. Results from the systolic hypertension in the elderly program. Arch Intern Med 1994;154: 2154–2160.
27. Forette F, Seux ML, Staessen JA, et al. Prevention of dementia in randomised double-blind placebo controlled Systolic Hypertension in Europe (Syst-Eur) trial. Lancet 1998;352:1347–1351.
28. Lithell H, Hansson L, Skoog I, et al. The Study on Cognition and Prognosis in the Elderly (SCOPE): principal results of a randomized double-blind intervention trial. J Hypertens 2003;21:875–886.
29. Saxby BK, Harrington F, Wesnes KA, McKeith IG, Ford GA. Candesartan and cognitive decline in older patients with hypertension: a substudy of the SCOPE trial. Neurology. 2008;70:1858-66
30.. Birns J, Morris R, Jarosz J, Markus HS, Kalra L. Hypertension-related cognitive decline: is the time right for intervention studies? Minerva Cardioangiol. 2009;61:813-830
31. Di Bari M, Pahor M, Franse LV, Shorr RI, Wan JY, Ferrucci L, Somes GW, Applegate WB. Dementia and disability outcomes in large hypertension trials: lessons learned from the Systolic Hypertension in the Elderly Program (SHEP) trial. American Journal of Epidemiology. 2001;153:72-78
32. Dichgans M, Markus HS, Salloway S, Verkkoniemi A, Moline M, Wang Q, Posner H, Chabriat HS. Donepezil in patients with subcortical vascular cognitive impairment: a randomised double-blind trial in CADASIL. Lancet Neurol. 2008;7:310-8
33. Fazekas F, Kleinert R, Offenbacher H et al. Pathologic correlates of incidental MRI white matter signal hyperintensities. Neurology 1993; 43: 1683-1689.
34. Scheltens P, Leys D, Barkhof F, Huglo D, Weinstein HC, Vermersch P, et al. Atrophy of medial temporal lobes on MRI in "probable" Alzheimer's disease and normal ageing: diagnostic value and neuropsychological correlates. Journal of Neurology, Neurosurgery & Psychiatry 1992; 55:967-972.
35. Strauss E, Sherman EMS, Spreen O. A Compendium of Neuropsychological Tests: Administration, Norms and Commentary. Oxford University Pres. 2006.

36. Wechsler D. The Wechsler Adult Intelligence Scale IV. Pearson Education Inc. 2008.
37. Nasreddine ZS, Phillips NA, Bédirian V, Charbonneau S, Whitehead V, Collin I, Cummings JL, Chertkow H. The Montreal Cognitive Assessment, MoCA: a brief screening tool for mild cognitive impairment. Am Geriatr Soc. 2005; 53: 695-9.
38. Van Der Elst W, Van Boxtel MPJ, Van Breukelen GJP. Rey's verbal learning test: Normative data for 1855 healthy participants aged 24-81 years and the influence of age, sex, education, and mode of presentation. J Int Neuropsychological Soc. 2005: 11 290-302.
39. Nelson HE. National Adult Reading Test, 2nd edition. NFER-Nelson Publishing Company: Berkshire, England. 1981
40. Gelinas I, Gauthier L, McIntyre MC, Gauthier S. Development of a functional measure for persons with Alzheimer's disease: The disability assessment of dementia. American Journal of Occupational Therapy. 1999; 53 471-478.
41. Williams LS, Weinberger M, Harris LE, Clark DO, Biller J. Development of a stroke-specific quality of life scale. Stroke. 1999;30:1362-9.
42. Brookes R, Willis TA, Patel B, Morris RG, Markus HS. Depression is a major predictor of quality of life in cerebral small vessel disease. (submitted)

Appendix 1:

Visit Time Windows

After randomisation:

- Baseline assessments & cognitive tests to be carried out within 7 days
- Baseline DTI MRI to be carried out within 14 days (if applicable)
- *For sites taking part in the Perfusion sub-study, Baseline scan **must** take place before treatment is changed*

Baseline to 1 month	1 calendar month +/- 7 days
Baseline to 3 months	3 calendar months +/- 7 days
Baseline to 6 months	6 calendar months +/- 14 days
Baseline to 12 months	12 calendar months +/- 14 days
Baseline to 18 months	18 calendar months +/- 14 days
Baseline to 24 months	24 calendar months +/- 14 days

eCRF Completion

- All data to be entered on the eCRF within 1 week of a visit
- Queries to be resolved within 2 weeks

Additional Data

- Copies of cognitive tests and questionnaires to be sent to the coordinating centre within 2 weeks of a visit
- Copies of MRI scans to be sent within 2 weeks of scan taking place

Appendix 2. Summary table of study assessments

**Study Procedures**	**Screening**	**Baseline**	**Follow up** ***(1 mo)***	**Follow up (3 mo)**	**Follow up** ***(6 mo)***	**Follow up (*12 mo*)**	**Follow up (*18 mo*)**	**Follow up (*24 mo*)**
Informed consent	X							
Inclusion/exclusion criteria	X							
Medical history	X							
Review brain scan	X							
Review of medication	X	X	X	X	X	X	X	X
Blood pressure	X	X	X	X	X	X	X	X
Neurological and cardiovascular examination		X						
Cognition		X		MOCA only		X		X
Disability scales		X				X		X
QOL scales		X				X		X
Structural MRI (If in substudy)		X						X
Perfusion MRI (If in substudy)		X		X				X
Venupuncture		X						

PRESERVE Baseline to 3 month ASL Analysis Plan

Key Question: Does intensive vs. standard treatment of blood pressure (bp) lead to a change in CBF over 3 months?

Main Analysis Objectives

Primary Outcome Variable

ANOVA - Change in whole brain CBF between main treatment groups.

Secondary Outcome Variables

1. ANOVA - Repeat the main analysis looking individually at grey matter (GM), white matter (WM) and normal-appearing white matter (NAWM) CBF as outcomes.

2. *t*-test/Mann Whitney U - Compare Adverse Events/SAE's between the two groups.

Further Sub-group Analyses

New patient groupings will be generated based those who achieved their bp target at 3 months (i.e. <125 systolic vs. >130 systolic as per the study targets). Repeat the above ANOVA analyses with these sub-groups.

Further Analyses

Linear regression – Change in bp vs. change in CBF.

The Analyses

General statistical bookkeeping

- Check distribution of all variables in main treatment and subgroups.
- Homogeneity of variance tests between variables after being split into groupings based on treatment / bp.
- Examinations to make sure the different experimental groups (both treatment arm and achieved bp groupings) are similar, i.e. groupwise testing to examine for any *baseline* differences in:
    - Age
    - Days from stroke to scan
    - Days between baseline and 3 month scan
    - bp
    - Number of bp medications
    - MoCA performance
- …and Chi-squared to look at differences in ratio-type variables:
    - Gender
    - Risk factors (previous stroke, history of cog. decline/apraxia/hypercholesterolaemia/depression, diabetes, smoker, angina, myocardial infarction on CABG, periphery vascular disease).
- Variability of change in CBF will be examined to identify if any outliers exist. A decision will then made be regarding if any data points should be excluded as outliers.

Looking at bp treatment success

- Report % of each group which succeeded in reaching their target bp.
- Paired *t*-tests to statistically examine bp change in each treatment group.
- Independent *t*-tests to statistically examine bp change between treatment groups.

Testing our main objectives

- Primary outcome of change in CBF between treatment groups will be tested using an ANOVA which also controls for site.
- Secondary outcomes of the different tissue ROI's will also be tested between treatment groups in an ANOVA, controlling for site.
- These tests will be repeated using the different groupings based on patients who "achieved" their bp (intensive= <125, standard= >130).
- Where appropriate after an ANOVA, post-hoc tests looking at exact means / hazard ratios etc. will be conducted. Levines Test of equal variances will also be used to see that ANOVA assumptions have been met. Analysis will be repeated using non-parametric testing when necessary.
- A regression model will use change in CBF as the DV and baseline / 3 month change in bp as a predictor variable to examine this relationship (while controlling for site).

- The distribution of residuals will be inspected for normality following regressions to ensure that regression assumptions have been met.
- The number of Adverse Events/SAE’s (deemed to have been “possible” or greater in their chance of being related to the study drug) will be compared between groups by independent *t*-tests / Mann Whittney U as appropriate.
